# Less is more: seipin, phospholipids, and embryogenesis

**DOI:** 10.1093/lifemeta/loac023

**Published:** 2022-09-17

**Authors:** Bin Liang, Jennifer L Watts

**Affiliations:** Center for Life Sciences, School of Life Sciences, Yunnan University, Kunming, Yunnan 650091, China; School of Molecular Biosciences, Washington State University, Pullman, WA 99164-7520, USA

**In a recent paper published in *Life Metabolism*, Zhu *et al.* revealed that mutations that reduce phosphatidylcholine (PC) synthesis rescue embryonic lethality but exacerbate lipid droplet (LD) abnormalities in *C. elegans seipin* mutants, demonstrating distinct roles for Seipin in embryogenesis and LD formation.**

Lipids serve vital roles as membrane constitutes, efficient energy storage molecules, and as precursors of potent cellular signals. Energy overload leads to the production of triacylglycerols (TAG) that are stored in LDs for later use. LDs are intracellular organelles present in some prokaryotes and almost all eukaryotes that dynamically regulate lipid and energy homeostasis. When energy and membrane synthesis is required, e.g. during division of rapidly growing cells, neutral lipids in LDs are released and converted to phospholipids and sphingolipids for growth and development. Therefore, cells must evolve exquisite mechanism to ensure appropriate lipid partitioning for life activities.

Seipin is an integral endoplasmic reticulum (ER) protein conserved from yeast to human. Seipin was initially identified as a critical factor involved in LD biogenesis and growth [1, 2]. In humans, Seipin deficiency causes Bernardinelli-Seip congenital lipodystrophy 2 (BSCL2)/congenital generalized lipodystrophy type II [3]. Numerous studies have focused on Seipin’s role in LD homeostasis. Meanwhile, several studies suggest that Seipin plays other roles. For example, dysfunction of Seipin causes muscular hypertrophy, mental retardation, and sperm abnormality in BSCL2 patients, as well as embryogenesis defects in *C. elegans* [4, 5]. These studies raised the question of how Seipin synergistically regulates LD homeostasis and other physiological processes, and whether there is a cause-and-effect between the abnormal LD homeostasis and physiological defects in *seipin*-deficient organisms.

*C. elegans* is a genetically tractable model organism that shares key genetic and physiological similarities with humans. SEIP-1 is the sole ortholog of human Seipin in *C. elegans*. A popular approach to identify biological mechanisms and alternative functions for conserved proteins is to employ genetic screens for new mutations that reverse specific phenotypes in a mutant strain. In this issue, Zhu *et al.* first found that the *seip-1(tm4221)* mutant displayed abnormal embryonic development and LDs compared to wild type (WT). Next, they performed a forward genetic screen to identify suppressors of the highly penetrant embryonic lethal phenotype of *seip-1(tm4221)* mutants. They found two suppressor lines, *xd286* and *xd287*, which were identified as mutations in the genes *spin-4* and *nhr-114,* respectively.

**Figure 1:**
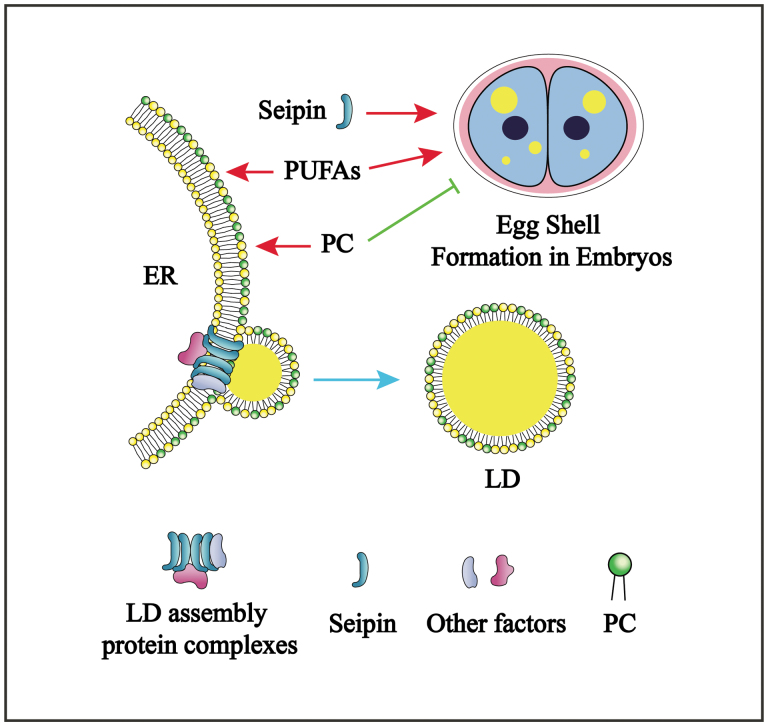
Seipin synergistically regulates LD growth and embryogenesis in *C. elegans*. PC deficiency suppresses the lethality, but enhances the LD abnormality in *seipin* mutants.

The nuclear hormone receptor NHR-114 was reported to act in the “B12-one-carbon cycle-PC” axis [6], and the SPIN-4 is an ortholog of the human lysosomal transporter SPNS1 [7]. Interestingly, Zhu *et al.* found that the *nhr-114(gk849)* and *spin-4(xd458)* mutants displayed similarities in response to B12 containing diet-induced sterility, suggesting they may function in the same pathway. Indeed, compared to the WT, both *nhr-114(gk849)* and *spin-4(xd458)* mutants have reduced level of B12, which could be rescued by dietary supplementation of B12, as well as methionine or choline. Furthermore, they uncovered that SPIN-4 acts upstream of NHR-114 to likely facilitate lysosomal B12 transport in the “one-carbon cycle-PC” pathway. Most importantly, they demonstrated that the level of PC in *seip-1(tm4221)* mutant embryos was slightly increased, and that reduction of PC synthesis by mutations of genes involved in the “B12-one-carbon cycle-PC” axis could suppress the embryonic lethality of *seip-1(tm4221)* mutants. In addition, knockdown of *epg-3*, the homolog of the VMP phospholipid scramblase, completely rescues the embryonic lethality of the *seip-1;spn-4* double mutant strain. Thus, phospholipid homeostasis in the ER is a key requirement for proper embryogenesis (Fig. 1).

Consistent with Seipin-deficient mutants of yeast, fly, and mouse, the *seip-1(tm4221)* mutants also display large LDs in mature oocytes. It is well known that either PC deficiency, or PC accumulation, leads to large LDs. Thus, the authors examined whether PC manipulation affects LD size in the *seip-1(tm4221)* mutant strain. They found that *spin-4(xd458)*, *nhr-114(xd428), metr-1(ok521),* and *pcyt-1(et9)* mutations, all of which affect PC levels, further increased the number of large LDs in *seip-1(tm4221)* oocytes. This enhancement of the *seip-1* phenotype, rather than suppression, suggests that PC homeostasis plays distinct roles in embryogenesis and in LD formation (Fig. 1).

The biological function of Seipin is still an enigma. The study of Zhu *et al.* reveals that Seipin and PC regulate embryogenesis and LD homeostasis through distinct mechanisms in *C. elegans*. PC deficiency recovers the embryonic lethality of *seipin* mutants, while it enhances the large LD phenotype. However, many questions remain. Recent studies examining LD formation reveal that Seipin monomers associate to form a “cage” that regulates TAG entry during LD formation [8]. How this structural function of Seipin relates to *C. elegans* embryogenesis is worthy of further study. While others have shown that *de novo* lipid biosynthesis and polyunsaturated fatty acids (PUFAs) are required for eggshell formation [5, 9, 10], this study reveals that precise levels of PC are a requirement. Further studies of lipid components necessary for the rapid synthesis of the eggshell and other barriers will shed light on roles of structural proteins such as Seipin in these important processes.
